# Pediatric Emergencies and Hospital Admissions in the First Six Months of the COVID-19 Pandemic in a Tertiary Children’s Hospital in Romania

**DOI:** 10.3390/children9040513

**Published:** 2022-04-05

**Authors:** Victor Daniel Miron, Deniz Gunșahin, Claudiu Filimon, Gabriela Bar, Mihai Craiu

**Affiliations:** 1 Carol Davila University of Medicine and Pharmacy, 050474 Bucharest, Romania; claudiu.filimon@stud.umfcd.ro (C.F.); mihai.craiu@umfcd.ro (M.C.); 2 National Institute for Mother and Child Health “Alessandrescu-Rusescu”, 020395 Bucharest, Romania; bargabriela@yahoo.com; 3 University Emergency Hospital, 050098 Bucharest, Romania; deniz_gunsahin@yahoo.com

**Keywords:** COVID-19, children, emergency, hospitalization, pediatric healthcare

## Abstract

The COVID-19 pandemic has had a significant impact on the pediatric population, particularly on their access to health services. We conducted a retrospective study to assess the influence that the pandemic, and its related containment and mitigation public health measures, had on pediatric emergencies and hospitalizations in a major tertiary pediatric hospital in Bucharest, Romania, during the first six months of the pandemic, March–August 2020, compared to the same period in 2019. In these first 6 months of the COVID-19 pandemic, the number of pediatric emergencies decreased 2.8-fold compared to the same period in 2019, but the proportion of major emergencies increased significantly (*p* < 0.001). The number of admissions also decreased 3.3-fold in 2020, compared to 2019, but the risk of admission for lower respiratory tract infections and respiratory failure increased 1.3- and 2.3-fold, respectively. In conclusion, the restrictions imposed by the pandemic containment and mitigation plan not only had a significant impact on reducing emergency department presentations, but also on pediatric admissions in Romania. These data highlight the importance of maintaining optimal access to child health services when confronted with a public health threat, such as the COVID-19 pandemic. Active communication with parents, involving general practitioners, pediatricians, and authorities, is essential for managing children with acute signs of illness in the case of future restrictions or lockdown measures.

## 1. Introduction

The emergence of severe acute respiratory syndrome coronavirus 2 (SARS-CoV-2) has taken the world by surprise. The novel virus of the *Coronaviridae* family spread rapidly across all continents [1]. Subsequently, drastic measures were taken in almost all countries, including Romania, in an effort to contain, or at least limit, the spread of SARS-CoV-2 [2]. In order to cope with the increasing caseloads of patients with coronavirus disease (COVID-19), hospitals were reorganized, so that in the first months of the pandemic, access to the healthcare system for non-COVID-19 patients (adults and children) was limited [3]. In addition, increased fear of possible infection with SARS-CoV-2 changed human attitudes. This fear was manifested at a higher intensity by parents when it came to their children [4].

Although lockdown played a significant role in reducing the circulation of SARS-CoV-2 and of other viruses in the pediatric population [5], many children still experienced a range of age-specific acute illnesses. However, the number of emergency department (ED) presentations and admissions was reported to have decreased significantly among children [6,7,8]. Telemedicine [4,9], or even self-medication, were, at times, preferred over a physical visit to the doctor [4]. Similar trends were observed among adults [10].

SARS-CoV-2 infection has spread rapidly in the adult population, putting enormous pressure on even the best-performing healthcare systems in highly developed countries [11,12,13,14]. In contrast, the magnitude and severity of pediatric COVID-19 cases have fortunately reduced [15,16], at least during the first pandemic waves. In Romania, the impact of the pandemic on the pediatric population has been poorly studied. According to the National Institute of Public Health, the rate of COVID-19 cases among children remained below 5% in both the 0–9 and 10–18 age groups, including during the circulation of subsequent variants of SARS-CoV-2, Delta and Omicron [17]. However, the impact of the pandemic should not only be considered in terms of the number of cases of SARS-CoV-2 infection. The effects of the pandemic, direct and indirect, are much more complex, and need to be understood as soon as possible to inform future interventions. The first 6 months of the pandemic had, by far, the most significant impact on people. During this period, a strict lockdown was instituted worldwide [18], including in Romania [2], and people’s fear of illness was increased [4,19].

Therefore, in order to understand the dynamics of the pandemic on children in our country, and to explore potential approaches and planning for future emergency events, we aimed to conduct a retrospective study on ED presentations and hospitalizations in the first 6 months of the COVID-19 pandemic, compared to the same period in 2019.

## 2. Materials and Methods

We conducted a retrospective analysis of all pediatric emergencies and cases hospitalized for acute pathologies in epidemiological weeks 10–35, corresponding to the years 2019 (3 March–31 August 2019) and 2020 (1 March–29 August 2020), in the “Alessandrescu-Rusescu” National Institute for Mother and Child Health (NIMCH), Bucharest, Romania.

The “Alessandrescu-Rusescu” NIMCH is a tertiary children’s hospital and an important pediatric research center in Bucharest, the capital of Romania. The hospital serves the population of the northern part of the capital and the neighboring Pipera-Voluntari areas, providing emergency care in the ED, as well as hospitalization, especially for acute respiratory, gastrointestinal, and renal pathologies.

In order to better assess the impact of the COVID-19 pandemic on pediatric pathologies during the initial lockdown period, we analyzed the data starting with the 10th epidemiological week of 2020, considering that the first case of COVID-19 was reported in Romania on 26 February 2020 [20,21], the World Health Organization declared a pandemic on 11 March 2020 [1], and a state of health emergency was declared in Romania on 16 March 2020 [2]. All data were compared with the similar period in the pre-pandemic year 2019.

In the analysis of pediatric emergencies, we included all pediatric patients (under 18 years old) who presented with an acute illness and were assessed in the ED during the two study time spans.

In the ED of the “Alessandrescu-Rusescu” NIMCH, all cases are classified by color codes, according to the type of emergency under the protocol of the Ministry of Health [22]. Thus, the white code represents “consultation”, the blue code “non-emergency”, the green code “minor emergency”, the yellow code “major emergency”, and the red code “resuscitation”. Data on date of presentation, age, gender, and type of emergency were collected from the hospital’s electronic system.

In the analysis of hospitalizations, we included all children under 18 years of age who received hospital care for at least one night for an acute illness during the two periods. The ICD-10 disease classification was used to select cases from the hospital electronic system. The following disease codes were included in the study: A02, A03, A04, A08, A09, K52, E86, E87, J00, J01, J02, J03, J04, J06, J10, J11, J12, J13, J17, J18, J20, J21, J22, J44, J45, J93, J96, R09, H60, H65, H66, H67, N39, N00, G01, G02, G03, A38, B15 and B08. Identified patients were analyzed and duplicates were excluded from the final analysis. Discharge diagnoses were categorized into the following 4 disease groups: respiratory disease, gastrointestinal disease, reno-urinary disease, and other acute disease (Supplementary Material). Patients with chronic diseases, hospitalized for reassessment or for chronic treatment, were excluded from the analysis. The following data were collected for each case: date of admission, age, gender, and duration of hospitalization.

Statistical analysis was performed with IBM SPSS Statistics for Windows, version 25 (IBM Corp., Armonk, NY, USA). The level of statistical significance was set at *p* < 0.05. For continuous variables, data normality was checked with the Shapiro–Wilk test. Because all continuous variables had non-Gaussian distributions, we reported the median along with the interquartile range (percentiles 25 and 75), as well as the results of the nonparametric Mann–Whitney U test, for comparative analysis. Effect size was calculated as described in the literature [23]. Categorical variables are reported as frequencies and percentages, and for comparative analysis, we used the chi-square test with odds ratio (OR) and a 95% confidence interval (95%CI).

## 3. Results

### 3.1. Analysis of Pediatric Emergencies

**Figure 1:**
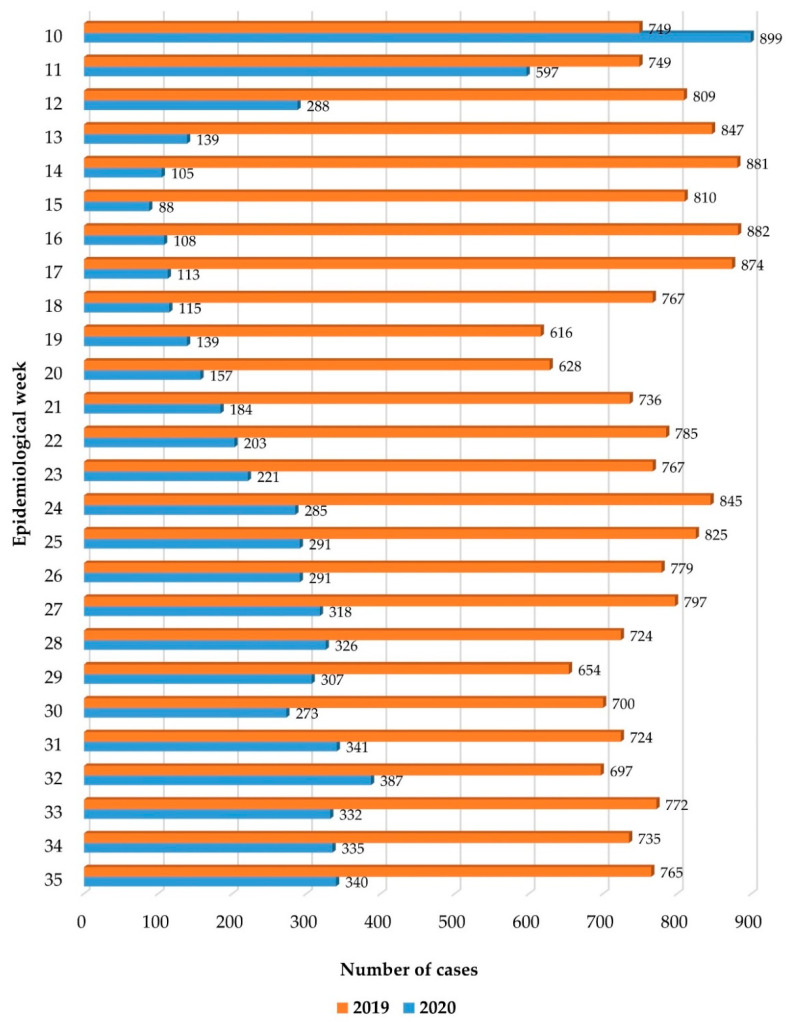
Distribution of pediatric cases presented to the emergency department by epidemiological weeks (2019 vs. 2020).

In the first 6 months of the COVID-19 pandemic, the number of pediatric emergencies decreased 2.8-fold compared to the same period in 2019 (20117 children in 2019 vs. 7291 children in 2020). Throughout 2019, the number of presentations per week had been steady, with an average of 745 presentations. In contrast, in 2020, once the state of emergency was established in Romania, the number dropped sharply from 899 presentations in week 10/2020 to 288 presentations in week 12/2020, reaching a historical low of 88 presentations in week 15/2020. Once the state of emergency was lifted in week 20/2020, the number of ED presentations started to increase, remaining relatively constant at around 300 cases per week (Figure 1).

In both years, males were predominant, in terms of the number of presentations (53.7%, *n* = 10812 in 2019; 52.5%, *n* = 3828 in 2020; *p* = 0.068). In 2020, the median age was significantly lower compared to 2019 (2.7 years (IQR: 1.1, 5.8) vs. 3.3 years (IQR: 1.3, 6.3); *p* < 0.001, U = 68441056.0, z = −6.074, r = 0.037).

Although minor emergencies accounted for the majority of presentations to the ED in both years, in 2020, these were significantly more frequent compared to 2019 (77.7% vs. 59.9%; *p* < 0.001). Similarly, major emergencies were also significantly more frequent in 2020 (2.1%, 152 cases) compared to the pre-pandemic year 2019 (1%, 195 cases; *p* < 0.001). In contrast, there were significantly more non-emergencies in 2019 compared to 2020 (20.2% vs. 39.1%; *p* < 0.001) (Table 1). In terms of median age by type of emergency, with the exception of minor emergencies, there were no statistically significant differences between patients in the two-time spans compared (Table 1).

### 3.2. Analysis of Hospitalized Cases

A total of 631 children were hospitalized for acute illness in the first 6 months of the COVID-19 pandemic, 3.3 times fewer compared to the same period in 2019 (*n* = 2058). The rate of hospitalizations for acute illness, relative to the number of ED presentations, was higher in 2019 (10.2% vs. 8.7%; *p* < 0.001, χ^2^ = 15.0, OR = 1.2, 95%CI = 1.1–1.3). In both of the studied time spans, male patients predominated (55.5%, *n* = 1137 male children in 2019; 56.7%, *n* = 355 in 2020). The median age of the patients admitted in 2020 was significantly higher compared to those admitted in 2019 (1.8 years (IQR: 0.6, 5.9 years) vs. 1.3 years (IQR: 0.5, 3.8 years); *p* < 0.001, z = −4.360, r = 0.1).

The spectrum of pathologies requiring hospitalization differed between the two periods analyzed. In 2020, patients with acute lower respiratory tract infections (LRI) and those with respiratory failure had 1.3-fold and 2.3-fold higher odds of hospitalization compared to 2019 (Table 2); while, in 2019, patients with acute upper respiratory tract infections (URI) and those with gastrointestinal disease had 1.9-fold and 1.3-fold higher odds of hospitalization (Table 2). By analyzing the median ages by the type of pathology for which they were admitted, we observed that in the pandemic year 2020, patients with gastrointestinal pathology and LRI had significantly higher ages compared to those in 2019 (Table 3).

The median length of hospital stay for 2020 was one day longer than in 2019 (4 days (IQR: 2, 6 days) vs. 3 days (IQR: 2, 5 days); *p* = 0.068). In no disease category was there a significant difference in length of hospitalization between the two study time spans analyzed (Table 4).

## 4. Discussion

The COVID-19 pandemic has had numerous implications for the pediatric population, but, in Romania, the data are mostly limited to the incidence, the overall number of cases, and their severity. The results of our study, presented above, play an important role in understanding this surprising emerging pandemic, and provide a true picture of the impact that the containment measures taken at the onset of the pandemic, along with the subsequent mitigation measures, had on the evolution of pediatric pathologies, and on the healthcare addressability of the pediatric population in Romania. The implications of the COVID-19 pandemic are wide ranging, and further studies are needed to fully understand the impact on children.

Globally, there has been a significant reduction in both ED presentations and admissions to pediatric hospitals, as we have also reported in this analysis. A number of studies have presented trends in their own countries, and have attempted to explain this phenomenon. Clearly, the lockdown measures taken at the onset of the pandemic, with very strict containment rules, have also led to a significant reduction in the circulation of other pathogens among children. Additionally, parents’ fears of a possible risk of infecting their children with SARS-CoV-2 if they present to the hospital contributed to the negative trends in addressability to pediatric health services.

In Italy, between March and April 2020, there was a significant decrease, by 67%, in the number of children assessed in the ED [24]. In Singapore, there was also a significant decrease, especially in presentations related to acute respiratory and gastrointestinal diseases [25]. In Brazil, the lockdown reduced ED presentations and admissions, especially among children under 5 years of age [26]. Similar data have been reported from the USA, UK and Israel [7,8,27,28]. In addition, as mentioned above, the strict containment measures imposed at the beginning of the pandemic also reduced the circulation of other viruses; thus, a sudden temporary halt in the circulation of influenza or respiratory syncytial virus [29,30,31], and a significant reduction in the circulation of adenovirus and rotavirus, were observed [32].

A reduced and/or delayed presentation to the hospital was also accompanied by an increase in the severity of cases. In our study, we identified a significant increase in minor and major emergencies, but also a higher rate of hospitalization among children with LRI and respiratory failure, compared to the pre-pandemic period, explainable, in part, by delayed presentation to the hospital/physician for fear of the pandemic. Similarly, Matera et al. showed that, in Italy, the number of major emergencies increased in the early part of the pandemic, compared to 2019, most likely due to delayed presentation to a physician [33]. Clearly, delay in specialist medical assessment is a major problem associated with increased morbidity and mortality [34]. Thus, from the data presented, raising parents’ awareness of the need to evaluate their child’s danger signs is crucial. Recognizing when children show signs of acute illness, even with an altered general condition, should prompt the need to access rapid medical intervention. This awareness will increase the adequacy of the addressability to medical care, and will facilitate the optimization of child care, even in the unfortunate scenario of a potential subsequent new lockdown.

In contrast, during the same period, pediatric telemedicine (video call or phone call) was more frequently used than in the pre-pandemic period, including in Romania [9,35,36], as a useful tool to provide medical guidance to a parent concerned by their child’s illness. In addition to the possibility of contacting the general practitioner or pediatrician, a free, 24-hour pediatric helpline is available in Romania, Peditel (https://www.peditel.ro/, accessed on 1 March 2022), to which the number of phone calls increased by about 40% in the first months of the pandemic, compared to the same period in 2019 [37].

Romania exited the COVID-19 emergency state in epidemiological week 21/2020. This change was accompanied by less stringent public health measures, aimed at mitigation, instead of containment, and this resulted in an increase in pediatric cases assessed at the ED, with the number stabilizing at around 250–300 consultations/week, less than half of what it was in 2019. The relaxation of restrictions in our country was gradual, but schools remained closed until autumn 2020, and many parents continued to work from home and to limit many of their children’s social contacts for fear of catching COVID-19 [4]. All this contributed to a reduced number of illnesses in children, and, thus, reduced ED presentations and admissions, even as isolation measures were relaxed.

Several challenges that impact parental behavior and attitudes to child-care could be predicted. It is mandatory to address these behavioral patterns, in order to decrease the direct and indirect emotional impact of a future pandemic-related event, especially for fragile age groups, such as teenagers, or underprivileged children [38]. It would be efficient to empower parents in a shared decision-making process via education (recognition of early danger signs, especially in high-risk children [39], medical and digital literacy [40], etc.).

Our data, compared with those from already published studies, show the similarity in behavior and perception that people, regardless of country, have adopted in the face of this new health threat. All these results create a complex picture of the phenomenon observed at the beginning of the pandemic.

This study, along with the other data published on this subject, teaches us some important lessons to consider in the event of a new pandemic or medical crisis. Physician involvement must be active, and communication with patients/parents must be at the forefront. Parents should be advised on recognizing the signs of seriousness for acute disease in their children, so that health services can be accessed when needed and in a timely manner. Pandemic preparedness of health systems has become essential, to ensure timely responses, correct triage of patients, and to recognize emergencies, and ensure early and adapted medical intervention, while also maintaining access to health services for patients with chronic conditions. In addition, authorities need to communicate clearly and reassuringly to reduce people’s fear and mistrust.

Our study has a number of limitations. Due to the retrospective nature of the study, we were unable to obtain data on the associated chronic conditions of children who presented to the ED. We were also unable to quantify clinical complaints at the time of presentation or factors that led to the decision of hospital admission. In addition, it would have been interesting to question parents directly about their reasons for choosing whether or not to present to the hospital, and about fears they had concerning SARS-CoV-2 infection and lockdown.

This study also has a set of strengths, providing the first set of data to characterize the impact that lockdown and other pandemic control measures had on the pediatric population attending our hospital.

## 5. Conclusions

The COVID-19 pandemic and the restrictions imposed have had a significant impact on the reduction in ED presentations, but also on admissions to a tertiary pediatric hospital in Romania’s capital in a time-dependent manner, with a significant gap between the trends of in-hospital versus out-patient care. Pandemic-associated factors generated a remarkably higher impact on admissions than on emergencies. Major emergencies were more frequent than their corresponding pre-pandemic rates, and the odds of an LRI requiring hospitalization increased. These data highlight the importance of maintaining optimal access to child health services when confronted with a public health threat, such as the COVID-19 pandemic. Active communication with parents, involving general practitioners, pediatricians, and authorities, is essential for managing children with acute signs of illness in the case of future restrictions or lockdown measures.

## Figures and Tables

**Table 1:** Variation in number of presentations and median age by type of emergency.

Emergency Type	Number of Presentations		Median Age	
	2019 *n* = 20,117	2020 *n* = 7291	2019 Median (IQR)	2020 Median (IQR)
Consultation (white code)	5 (0.02%)	4 (0.05%)	NA	NA
	NA		NA	
Non-emergency (blue code)	7854 (39.1%)	1466 (20.2%)	3.3 (1.6, 5.8)	3.3 (1.4, 6.6)
	*p* < 0.001, χ^2^ = 854.9, OR = 2.5, 95%CI:2.4–2.7		*p* = 0.543	
Minor emergency (green code)	12048 (59.9%)	5664 (77.7%)	2.8 (1.0, 5.7)	2.5 (1.0, 5.7)
	*p* < 0.001, χ^2^ = 741.3, OR = 2.3, 95%CI:2.2–2.5		*p* < 0.001, z = −4.285, r = 0.032	
Major emergency (yellow code)	195 (1.0%)	152 (2.1%)	2.2 (0.9, 4.2)	1.8 (0.6, 4.0)
	*p* < 0.001, χ^2^ = 53.3, OR = 2.2, 95%CI:1.8–2.7		*p* = 0.550	
Resuscitation (red code)	5 (0.02%)	5 (0.06%)	NA	NA
	NA		NA	

NA—not applicable.

**Table 2:** Statistical analysis of the proportion of pathologies admitted during the two periods analyzed.

Disease Category	2019 *n* = 2058	2020 *n* = 631	Statistical Analysis
Gastrointestinal disease, *n* (%)	710 (34.5)	187 (29.6)	*p* = 0.023, χ^2^ = 5.1, OR = 1.3, 95%CI:1.1–1.5
Upper respiratory tract infection, *n* (%)	602 (29.3)	111 (17.6)	*p* < 0.001, χ^2^ = 33.7, OR = 1.9, 95%CI:1.5–2.4
Lower respiratory tract infection, *n* (%)	570 (27.7)	211 (33.4)	*p* = 0.005, χ^2^ = 7.7, OR = 1.3, 95%CI:1.1–1.6
Respiratory failure, *n* (%)	34 (1.7)	23 (3.6)	*p* = 0.002, χ^2^ = 9.2, OR = 2.3, 95%CI:1.3–3.9
Reno-urinary disease, *n* (%)	105 (5.1)	53 (8.4)	*p* = 0.002, χ^2^ = 9.5, OR = 1.7, 95%CI:1.2–2.4
Other acute diseases, *n* (%)	37 (1.8)	46 (7.3)	*p* < 0.001, χ^2^ = 48.7, OR = 4.3, 95%CI:2.8–6.7

**Table 3:** Median age by pathology and period analyzed.

Disease Category	2019 Median (IQR)	2020 Median (IQR)	Statistical Analysis
Gastrointestinal disease	0.8 years (0.4, 1.3)	1.3 years (0.6, 2.6)	*p* = 0.001, z = −5.05
Upper respiratory tract infection	2.3 years (0.8, 5.3)	2.1 years (1.0, 4.5)	*p* = 0.998
Lower respiratory tract infection	1.8 years (0.4, 6.1)	3.0 years (0.4, 8.6)	*p* = 0.017, z = −2.39
Respiratory failure	4.4 years (2.0, 7.4)	3.9 years (2.3, 8.6)	*p* = 0.942
Reno-urinary disease	1.0 years (0.4, 3,5)	0.9 years (0.4, 3.4)	*p* = 0.380
Other acute diseases	NA	NA	NA

**Table 4:** Median duration of hospitalization by pathology and period analyzed.

Disease Category	2019 Median (IQR)	2020 Median (IQR)	Statistical Analysis
Gastrointestinal disease	3 days (2, 5)	3 days (2, 5)	*p* = 0.496
Upper respiratory tract infection	3 days (2, 5)	3 days (1, 5)	*p* = 0.065
Lower respiratory tract infection	3 days (1, 6)	4 days (1, 6)	*p* = 0.058
Respiratory failure	3 days (2, 4)	4 days (3, 5)	*p* = 0.268
Reno-urinary disease	5 days (3, 6)	6 days (4, 7)	*p* = 0.054
Other acute diseases	NA	NA	NA
All patients	3 days (2, 5)	4 days (2, 6)	*p* = 0.068

## Data Availability

The datasets generated and analyzed during the current study are available from the corresponding author upon reasonable request.

## Supplementary Materials

The following supporting information can be downloaded at https://www.mdpi.com/article/10.3390/children9040513/s1: Table S1: distribution of ICD-10 codes by disease categories.
