# Supplementary material for: Pediatric Emergencies and Hospital Admissions in the First Six Months of the COVID-19 Pandemic in a Tertiary Children’s Hospital in Romania
Source: Children (Basel). 2022 Apr 5;9(4):513. doi: 10.3390/children9040513 (PMC9028540; doi:10.3390/children9040513)
Supplement: Supplementary file 1 [file children-09-00513-s001.zip › children-1642995-supplementary.pdf]

Supplementary material

Table S1. Distribution of ICD-10 codes by disease categories.

Disease category	ICD-10 code
Gastrointestinal disease	A02, A03, A04, A08, A09, K52, E86
Upper respiratory tract infection	J00, J01, J02, J03, J04, J06, J10, H60, H65, H66, H67
Lower respiratory tract infection	J11, J12, J13, J17, J18, J20, J21, J22, J44, J45
Respiratory failure	J93, J96, R09
Reno-urinary disease	N39, N00, N04
Other acute diseases	E86, E87, G01, G02, G03, A38, B15, B08
